# Reconstruction and Analysis of a Genome-Scale Metabolic Model of *Ganoderma lucidum* for Improved Extracellular Polysaccharide Production

**DOI:** 10.3389/fmicb.2018.03076

**Published:** 2018-12-11

**Authors:** Zhongbao Ma, Chao Ye, Weiwei Deng, Mengmeng Xu, Qiong Wang, Gaoqiang Liu, Feng Wang, Liming Liu, Zhenghong Xu, Guiyang Shi, Zhongyang Ding

**Affiliations:** ^1^Key Laboratory of Carbohydrate Chemistry and Biotechnology, Ministry of Education, School of Biotechnology, Jiangnan University, Wuxi, China; ^2^National Engineering Laboratory for Cereal Fermentation Technology, Jiangnan University, Wuxi, China; ^3^Key Laboratory of Cultivation and Protection for Non-Wood Forest Trees, Ministry of Education, College of Life Science and Technology, Central South University of Forestry and Technology, Changsha, China; ^4^School of Food and Biological Engineering, Jiangsu University, Zhenjiang, China

**Keywords:** *Ganoderma lucidum*, extracellular polysaccharide, genome-scale metabolic model, biosynthetic pathway, phenylalanine, simulation

## Abstract

In this study, we reconstructed for the first time a genome-scale metabolic model (GSMM) of *Ganoderma lucidum* strain CGMCC5.26, termed model *i*ZBM1060, containing 1060 genes, 1202 metabolites, and 1404 reactions. Important findings based on model *i*ZBM1060 and its predictions are as follows: (i) The extracellular polysaccharide (EPS) biosynthetic pathway was elucidated completely. (ii) A new fermentation strategy is proposed: addition of phenylalanine increased EPS production by 32.80% in simulations and by 38.00% in experiments. (iii) Eight genes for key enzymes were proposed for EPS overproduction. Model *i*ZBM1060 provides a useful platform for regulating EPS production in terms of system metabolic engineering for *G. lucidum*, as well as a guide for future metabolic pathway construction of other high value-added edible/ medicinal mushroom species.

## Introduction

*Ganoderma lucidum* (lingzhi or reishi mushroom) is a species well known for its edible and medicinal properties, and has a long history of use for prevention and treatment of various human diseases. Extracellular polysaccharides (EPSs) from *G. lucidum* comprise a structurally diverse group of macromolecules that display immunomodulatory, antitumor, and a wide range of other biological activities (Ferreira et al., 2015). Many studies have described enhancement of EPS production through optimization of medium and culture conditions in submerged fermentation (Tang and Zhong, 2003). However, EPS molecules, their structural features, and their biosynthetic pathways are all highly complex, and attempts to improve EPS production are often hampered by this complexity. There is an urgent need for more extensive, systematic knowledge of physiological features and metabolism of *G. lucidum* EPSs.

Genome-scale metabolic models (GSMMs), in which a systems biology approach is used to integrate genomic, transcriptomic, proteomic, and metabolomic data, are highly effective tools for metabolism research. GSMMs have been widely used for analysis of network properties, prediction of growth phenotypes, and interpretation of experimental data, particularly in *Escherichia coli* and *Saccharomyces cerevisiae* models (Kim et al., 2017).

There have been no reports to date of GSMMs for edible/ medicinal mushroom species. The publication in 2012 of the whole genome sequence of *G. lucidum* strain CGMCC5.26 (Chen et al., 2012), and subsequent related reports, have made GSMM reconstruction feasible for this species. Such reconstruction will help clarify *G. lucidum* global metabolism, guide design of metabolic regulation strategies, and indicate useful research targets of “wet” experiments. Biosynthetic pathways of EPSs remain poorly known at this point because of our inadequate knowledge of related enzymes and their functions. Adequate knowledge will require gene cloning and genetic transformation studies (Wang et al., 2017).

We describe here reconstruction of the first GSMM of *G. lucidum*, model *i*ZBM1060, and its application to elucidate detailed physiological characteristics and production of EPSs in this species. The nucleoside sugar biosynthetic pathway of model *i*ZBM1060 was elucidated completely, the reactions of this pathway are summarized and illustrated, and related strategies for improving EPS production are proposed.

## Materials and Methods

### Reconstruction and Refinement of *G. lucidum* GSMM

The availability of the whole genome sequence of *G. lucidum* allowed us to perform GSMM reconstruction according to a three-step general workflow scheme described previously (Thiele and Palsson, 2010).

**FIGURE 1:**
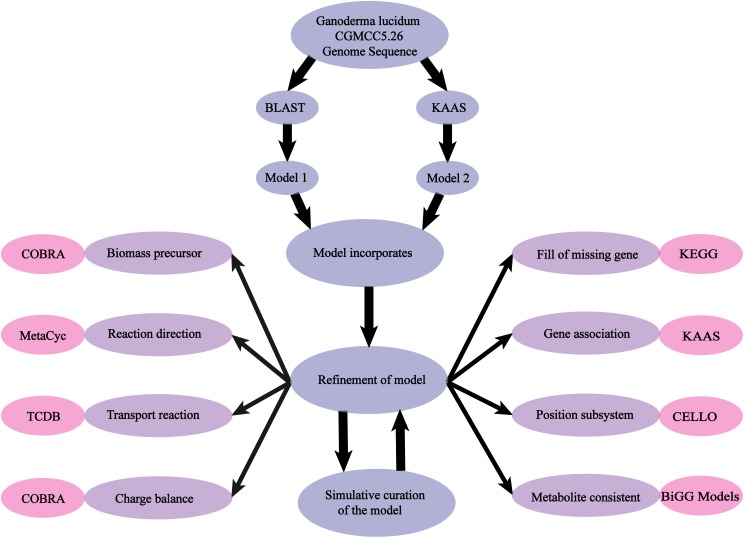
Scheme of model reconstruction process used in this study. Blue: basic process of model reconstruction. Purple: major aspects of refinement of the model. Pink: usage of Database and Toolbox to refine the process.

(i) Sequenced *G. lucidum* genome data were downloaded from the UniProt database (UniProt, 2010). Genes were functionally annotated by two methods: (a) Thresholds of the bidirectional BLAST for a functional sequence were set to have *e*-value < 1 × 10^−30^, amino acid sequence identity > 40%, and matching length ≥ 70% of the query sequence (Liu et al., 2012). An original reactions list was produced by selecting GSMMs of *Aspergillus niger i*MA871(Andersen et al., 2008), *Mortierella alpina i*CY1106 (Ye et al., 2015), and *Aspergillus terreus i*JL1454 (Liu, et al., 2013) as template frameworks to map the assigned genes. (b) The KEGG Automatic Annotation Server (KAAS) (Moriya et al., 2007) was used for functional annotation of all amino acid query sequences.

(ii) A draft model was developed and used as a starting point for subsequent network refinements. Biochemical information was acquired from public databases [KEGG (Kanehisa et al., 2010), MetaCyc (Caspi et al., 2008), CELLO (Yu et al., 2004), and TCDB (Saier et al., 2006)], and manual revisions (deletion of error reactions, addition of organism-specific information, checking of mass-charge balance, filling of metabolic gaps) were conducted sequentially.

(iii) The COBRA Toolbox was used to simulate growth rate and product formation, and the model was validated by comparison of results with experimentally observed phenotypes (Figure 1).

### Biomass Composition and Determination of Target Equation

The biomass components of *G. lucidum* are proteins, DNA, RNA, lipids, glucan, chitin and small molecules. Detailed information on biomass composition is summarized in Supplementary Table S1. A metabolic model (Andersen et al., 2008) were used as reference to calculate ATP required for cell growth and RNA: DNA ratio. Nucleotide and amino acid compositions were calculated based on *G. lucidum* genome (Chen et al., 2012). Detailed compositions of individual macromolecules were derived from published reports on *G. lucidum* (Mau et al., 2001; Stojkovic et al., 2014). A target equation of EPS production was determined based on mole percentages of monosaccharides in EPSs (Peng et al., 2015).

### *G. lucidum* Strain and Culture Conditions

*Ganoderma lucidum* CGMCC5.26 was obtained from the China General Microbiological Culture Collection Center (Beijing) and maintained on potato dextrose agar slants at 4°C. The seed and fermentation medium [glucose 20 g/L, yeast nitrogen base without amino acids (YNB) 5 g/L, tryptone 5 g/L, KH_2_PO_4_ 4.5 g/L, MgSO_4_⋅7H_2_O 2 g/L, initial pH 6.0] was kept at 30°C on a rotary shaker (150 rpm). The minimal growth medium for functional tests was composed of carbon source 20 g/L, nitrogen source 10 g/L, KH_2_PO_4_ 4.5 g/L, MgSO_4_⋅7 H_2_O 2 g/L, initial pH 6.0.

### Determination of Biomass, Residual Sugar in Medium, and EPS

Mycelia were harvested by centrifugation (10,000 rpm) for 10 min. The precipitate was washed three times with distilled water, and dried at 60°C to constant weight. Dry weight (DW) was determined by gravimetric method. Amount of residual sugar in medium was determined by 3, 5-dinitrosalicylic acid (DNS) method (Dubois et al., 1956).

For determination of EPS, centrifugal fluid as above was precipitated with adding 4 times of 95% (v/v) ethanol and left 8 h at 4°C to precipitate crude polysaccharides. Precipitate was collected by centrifugation (8,000 rpm) for 20 min, washed three times with 80% (v/v) ethanol, and dried at 60°C to remove residual ethanol. Total EPS content was assayed by phenol-sulfuric acid method (Dubois et al., 1951).

### Simulation, Curation, and Analysis of Model *i*ZBM1060

To assess the ability of the reconstruction to accurately reflect metabolic processes of *G. lucidum*, we converted the reaction list to a standard SBML document that could be read by COBRA Toolbox (Schellenberger et al., 2011) and subjected to Flux balance analysis (FBA) (Lakshmanan et al., 2014). Flux ranges of reactions in the network were limited for simulations (Thiele and Palsson, 2010). Essential elements must be obtained from the environment through the exchange reaction (Wang et al., 2016). For growth simulation, the biomass equation in minimal medium (no amino acids) was set as the objective function. A complex fermentation medium (basic elements and 20 amino acids) was simulated for EPS production, and maximal uptake rate for each amino acid was set to 0.01 mmol/gDW/h (Ye et al., 2015). Essential genes were assessed by setting fluxes of reactions to zero, and simulating optimal growth rate with FBA. The criterion for an essential gene was that its deletion results in zero growth.

For identification of target genes, MOMA (Segre et al., 2002) framework was used for better prediction of flux distribution. The overexpression algorithm involved five steps (Boghigian et al., 2012): (i) EPS production flux was imposed on the reconstructed model. (ii) Flux for each reaction was calculated based on the fermentation medium. (iii) Amplification of flux was imposed on individual reactions with non-zero flux, to simulate the effect of gene overexpression. (iv) MOMA was performed to overcome the problem of overexpression. (v) An overexpressed target having higher EPS production and f_PH_ value > 1 was identified (Equation 1), f_PH_ being the product of the specific biomass overexpression and specific EPS overexpression rates.

(1) $$\begin{array}{lll} f_{\mathrm{PH}} & = & =(f_{\mathrm{biomass}})(f_{\mathrm{EPS}})\\ & = & \left(\frac{V_{\mathrm{biomass, overexpression}}}{V_{\mathrm{biomass, WT}}}\right)\left(\frac{V_{\mathrm{EPS, overexpression}}}{V_{\mathrm{EPS, WT}}}\right) \end{array}$$

## Results and Discussion

### Reconstruction and Characteristics of Model *i*ZBM1060

**FIGURE 2:**
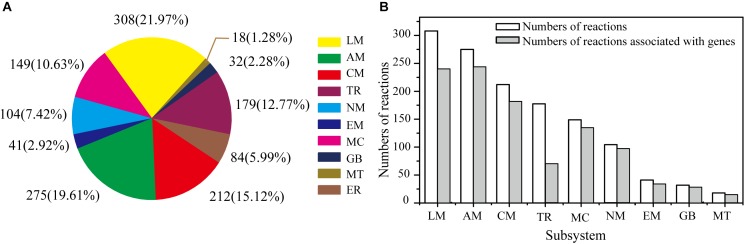
Properties of model *i*ZBM1060. **(A)** Numbers of reactions in each subsystem, and corresponding percentages of total reactions. **(B)** Numbers of reactions associated with genes in each subsystem. LM, lipid metabolism; AM, amino acid metabolism; CM, carbohydrate metabolism; TR, transport reactions; MC, metabolism of cofactors and vitamins; NM, nucleotide metabolism; EM, energy metabolism; GB, glycan biosynthesis and metabolism; MT, metabolism of terpenoids and polyketides; ER, exchange reactions.

The GSMM reconstruction was completed by automatic annotation and manual refinement, and a reaction list was obtained through KAAS and BLASTP. The final reconstructed GSMM of *G. lucidum*, termed model *i*ZBM1060, contained 1060 genes, 1202 metabolites, and 1404 reactions (Supplementary Table S2). The 1404 reactions in model *i*ZBM1060 were classified into 10 subsystems, according to the KEGG Pathway Database (Figure 2A). The largest subsystem (accounting for 21.97% of the 1404 reactions) was lipid metabolism (fatty acid biosynthesis; fatty acid degradation; glycerolipid, glycerophospholipid, sphingolipid, and steroid metabolism), followed by amino acid metabolism and carbohydrate metabolism. These three subsystems, combined, accounted for >50% of the 1404 reactions. There were a total of 1047 gene-associated reactions. In eight of the 10 subsystems, >80% of the reactions were associated with genes (the exceptions were lipid metabolism and transport reactions; Figure 2B).

### Growth Verification and Simulation in Model *i*ZBM1060

#### Qualitative Verification and Analysis of Growth Phenotypes

**FIGURE 3:**
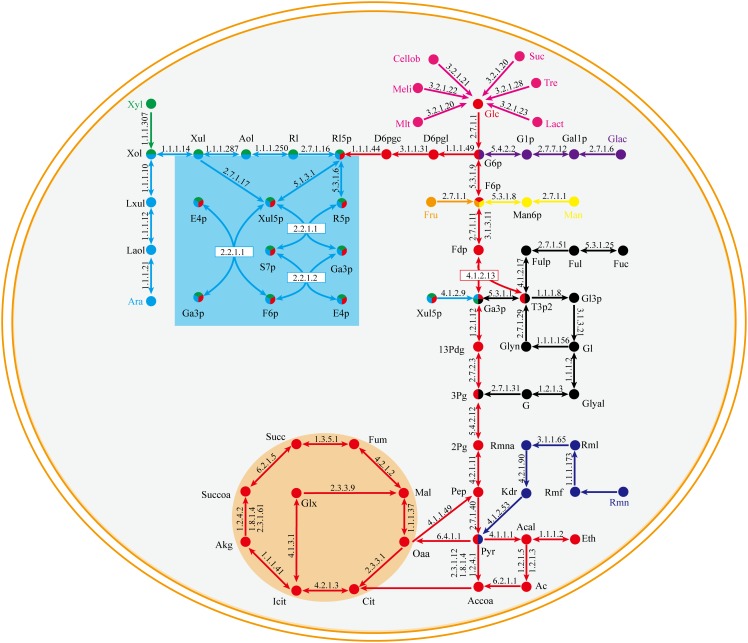
Central metabolic pathway of representative carbon sources in model *i*ZBM1060 (detailed information on enzyme information and reactions are presented in Supplementary Table S3).

The central metabolic pathway of *G. lucidum* carbon sources is shown schematically in Figure 3. Glucose, galactose, mannose, and fructose produce a corresponding phosphate monosaccharide through action of a kinase, and the monosaccharide then passes directly into the tricarboxylic acid (TCA) cycle, glyoxylate cycle, and pentose phosphate pathway (PPP). Xylose and arabinose are first phosphorylated by oxidation-reduction reaction, and then fructose-6-phosphate (fructose-6-P) is synthesized. In rhamnose and fucose metabolism, pyruvate and glycerone phosphate (respectively) are synthesized firstly.

The capability of *G. lucidum* to utilize 18 different carbon sources (13 saccharides, 3 alcohols, 2 carboxylic acids) for cell growth was predicted qualitatively by FBA. Each of the carbon sources was used as sole carbon source in minimal growth medium. Results were compared to experimental data, and the growth phenotype matching rate was 94.4%(Table 1). *G. lucidum* is able to utilize not only glucose, galactose, mannose, arabinose, xylose, rhamnose, fucose, and other monosaccharides, but also sucrose, maltose, lactose, and other disaccharides. FBA also predicted the capability to utilize various nitrogen sources (nitrate, urea, 20 amino acids) for cell growth. When results were compared to experimental data, the matching rate was 95.5% (Table 2).

**Table 1:** Growth phenotypic validation under a sole carbon source.

Substrate	Biomass		

Carbon source	*In vivo*	*In silico*	Reference
Glucose	+	+	Babitskaya et al., 2005; Wei et al., 2016
Sucrose	+	+	Babitskaya et al., 2005; Wei et al., 2016
Galactose	+	+	Babitskaya et al., 2005; Wei et al., 2016
Mannose	+	+	Babitskaya et al., 2005; Wei et al., 2016
Xylose	+	+	Babitskaya et al., 2005; Wei et al., 2016
Maltose	+	+	Babitskaya et al., 2005; Wei et al., 2016
Lactose	+	+	Babitskaya et al., 2005; Wei et al., 2016
Fructose	+	+	Babitskaya et al., 2005
Arabinose	+	+	Babitskaya et al., 2005
Mannitol	+	+	Babitskaya et al., 2005
Cellobiose	+	+	Babitskaya et al., 2005
Starch	+	+	Babitskaya et al., 2005
Fucose	+	+	Babitskaya et al., 2005
Rhamnose	+	+	This study
Inositol	−	−	This study
Ethanol	+	+	This study
Citrate	−	+	This study
Malate	+	+	This study

*In vivo, experimental results. In silico, simulation results.*

**Table 2:** Growth phenotypic validation under a sole nitrogen source.

Substrate	Biomass		

Nitrogen source	*In vivo*	*In silico*	Reference
Urea	−	+	This study
NH4Cl	+	+	This study
L-Methionine	−	−	This study
L-Isoleucine	+	+	This study
L-Leucine	+	+	This study
L-Phenylalanine	+	+	This study
L-Proline	+	+	This study
L-Alanine	+	+	This study
L-Glutamate	+	+	This study
L-Glutamine	+	+	This study
L-Glycine	+	+	This study
L-Threonine	+	+	This study
L-Aspartate	+	+	This study
L-Asparagine	+	+	This study
L-Tryptophan	+	+	This study
L-Histidine	+	+	This study
L-Serine	+	+	This study
L-Tyrosine	+	+	This study
L-Valine	+	+	This study
L-Lysine	+	+	This study
L-Arginine	+	+	This study
L-Cysteine	+	+	This study

*In vivo, experimental results. In silico, simulation results.*

*Ganoderma lucidum* grew successfully on 17 of the 18 carbon sources and 20 of the 22 nitrogen sources as above, indicating its broad substrate adaptability. There were no “fatal gaps” in model *i*ZBM1060, and it can therefore be used for predicting catabolic pathways of various carbon and nitrogen sources. Two of the apparently non-conforming sources (citrate and urea) can be attributed to unclear transport pathways and the absence of regulatory mechanisms in this stoichiometric model.

**FIGURE 4:**
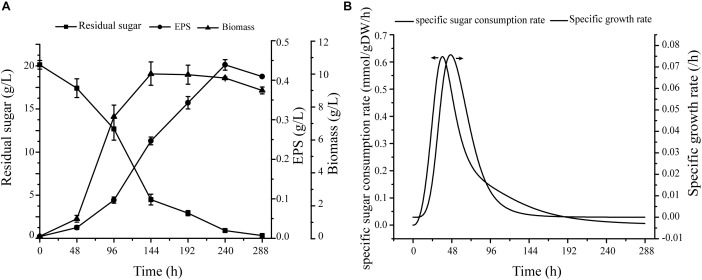
Verification of specific growth rates in cells. **(A)** Under conditions of fermentation medium, cell growth, EPS production, and sugar consumption curve. **(B)** Nonlinear curve fit of growth curve and sugar consumption curve.

On the basis of carbon source metabolic pathways and the experimental results, we selected seven monosaccharides as single carbon sources for evaluation of effects of various carbon sources on biomass and EPS production (Supplementary Figure S1 and Figure 4A). The consistency of results further supports the validity of the model.

#### Quantitative Verification

Fermentation data were used as constraints for simulation of cell growth, including specific growth rate and glucose uptake rate. Maximal specific growth rate was 0.076 h^−1^, and corresponding sugar consumption rate was 0.506 mmol/gDW/h (Figure 4B). For simulation of cell growth in various media, the biomass equation was maximized in flux analysis. For glucose medium without production constraints, predicted cell growth rate was 0.077 h^−1^ – only 1.3% higher than experimental growth rate (0.076 h^−1^).

#### Identification and Analysis of Essential Genes for Cell Growth

**FIGURE 5:**
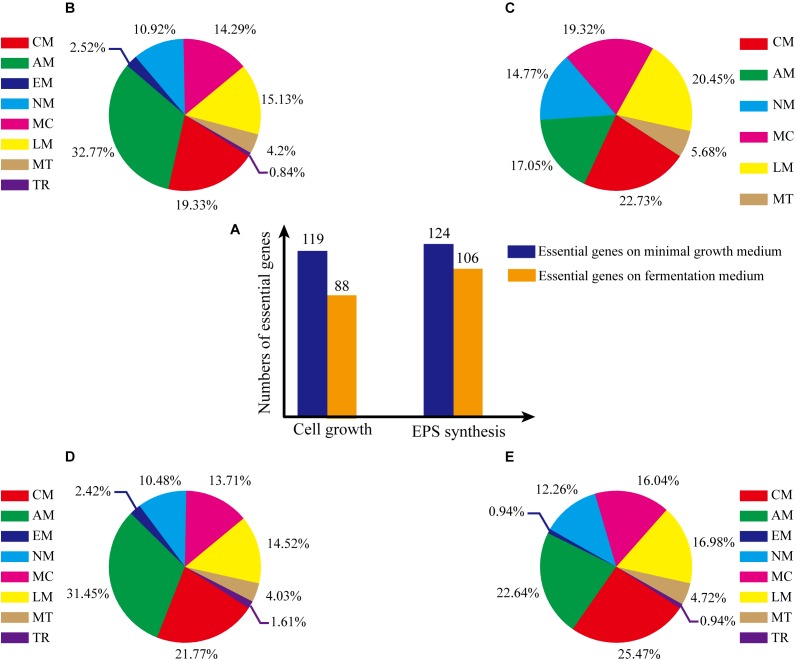
Percentages of essential genes in each subsystem under various culture conditions. **(A)** Numbers of essential genes under various conditions. **(B)** Essential genes for cell growth, on minimal growth medium. **(C)** Essential genes for cell growth, on fermentation medium. **(D)** Essential genes for EPS production, on minimal growth medium. **(E)** Essential genes for EPS production, on fermentation medium. The abbreviations of each subsystem are the same with that in Figure 2.

Consistency of growth rate *in silico* and *in vivo* indicated that model *i*ZBM1060 successfully reflected *G. lucidum* cellular metabolism. Essential genes for cell growth were predicted by single-gene deletion in COBRA Toolbox (MATLAB package) with two media (minimal growth medium, fermentation medium). Hundred and nineteen genes (11.23% of 1060 total genes) were predicted to be essential in minimal growth medium, and 88 genes (8.30% of total) were predicted to be essential in fermentation medium (Figure 5A). On minimal growth medium, >50% of the essential genes for growth were involved in either amino acid (32.77%) or carbohydrate metabolism (19.33%) (Figure 5B). In contrast, on fermentation medium, >90% of essential genes for growth were classified in 5 subsystems (amino acid metabolism, metabolism of cofactors and vitamins, nucleotide metabolism, carbohydrate metabolism, lipid metabolism) (Figure 5C), reflecting the important roles of these subsystems in cell growth (essential genes and simulation conditions are listed in Supplementary Table S4).

### Nucleoside Sugar Biosynthetic Pathway in *G. lucidum*

#### Construction of Nucleoside Sugar Biosynthetic Pathway

The biosynthetic pathway of EPSs can be divided into three stages: (i) biosynthesis of nucleoside sugar precursors; (ii) assembly of repeating units; (iii) process of polymerization (Li et al., 2016). The monosaccharide composition of all EPSs includes glucose, galactose mannose, xylose, arabinose, fucose, and rhamnose. Typically, the proportion of glucose is high whereas that of fucose and rhamnose is low (Peng et al., 2015; Wang et al., 2017). Monosaccharide heterogeneity is reflected in the complexity of EPS biosynthetic pathways.

Biosynthetic pathways of EPSs are poorly known because of our inadequate knowledge of related enzymes and their functions. On the basis of model *i*ZBM1060, we hereby propose a detailed nucleoside sugar biosynthetic pathway. Glucose, galactose, fucose, mannose, and arabinose reactions are catalyzed by monosaccharide kinase to produce corresponding phosphate monosaccharides, and UDP-glucose, UDP-galactose, GDP-mannose, and UDP-arabinose are then synthesized through action of pyrophosphorylase. E.g., hexokinase (GL26783-R1, GL20491-R1, and GL20491-R2) and mannose phosphomutase (GL20742-R1 and GL21817-R1) participate respectively in synthesis of mannose-6-P and mannose-1-P. GDP-mannose is then synthesized by action of GDP-mannose pyrophosphorylase (GL25424-R1).

**FIGURE 6:**
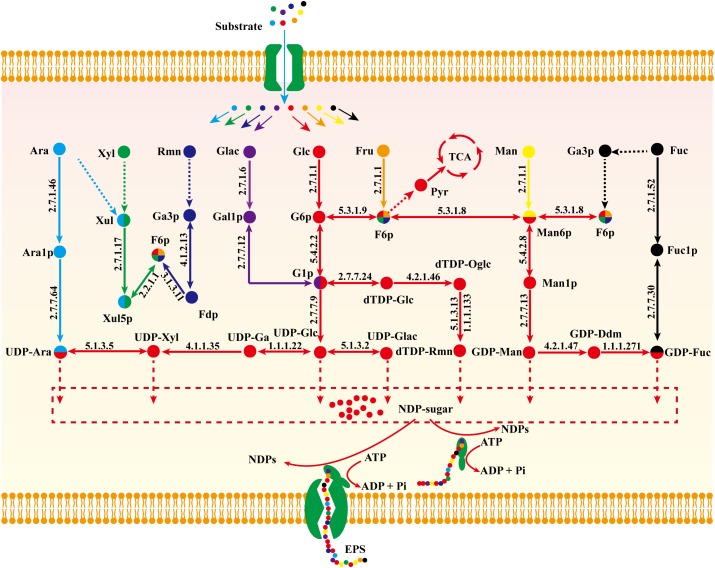
Proposed nucleoside sugar biosynthetic pathway of *G. lucidum* based on model *i*ZBM1060 (detailed information on enzyme information and reactions are presented in Supplementary Table S3).

Xylose enters the PPP to synthesize fructose-6-P, and then a nucleoside precursor. Fucose and rhamnose can also synthesize fructose-6-P via the gluconeogenesis pathway. Fructose-6-P has two pathways for synthesis of nucleoside sugar: (i) glucose-6-P isomerase (GL22245-R1) catalyzes conversion of fructose-6-P to glucose-6-P; (ii) fructose-6-P is converted to mannose-6-P by mannose-6-P isomerase (GL17878-R1 and GL22193-R1) and further synthesizes GDP-mannose and GDP-fucose (Figure 6).

Glucose, galactose, mannose, fucose, and arabinose are able to synthesize nucleoside sugars via short metabolic pathways. In contrast, xylose, fructose, and rhamnose cannot directly enter the nucleoside sugar biosynthetic pathway, and are therefore less ideal carbon sources. This concept is supported by our “wet” experimental results (Supplementary Figure S1 and Figure 4A).

The proposed nucleoside sugar biosynthetic pathway involves 20 genes, 17 enzymes, and glucose as carbon source. Peng et al. (2015) observed activity of related enzymes in a biosynthetic pathway, indicating the accuracy of our reconstructed pathway (Table 3).

**Table 3:** The reported enzymes of *G. lucidum* EPS biosynthetic pathway (Peng et al., 2015).

EC No.	Gene ID	Enzyme name	Reaction
5.3.1.9	GL22245-R1	Glucose-6-phosphate isomerase (GPI)	D-Glucose-6-phosphate <=> D-Fructose-6-phosphate
3.1.3.11	GL24591-R1 GL24591-R2	Fructose-1,6-bisphosphatase (FBP)	D-Fructose-1,6-bisphosphate - > D-Fructose-6-phosphate
5.3.1.8	GL17878-R1 GL22193-R1	Mannose-6-phosphate isomerase (PMI)	D-Mannose-6-phosphate <=> D-Fructose-6-phosphate
1.1.1.22	GL18437-R1	UDP-glucose-6-dehydrogenase (UGDH)	UDP-glucose <=> UDP-glucuronate
5.4.2.2	GL24280-R1	Glucose phosphomutase (PGM)	D-Glucose 1-phosphate <=> D-Glucose-6-phosphate
2.7.7.9	GL25739-R1	UTP-glucose pyrophosphorylase (UGP)	D-Glucose-1-phosphate - > UDP-glucose
2.7.7.24	Not annotated	dTDP-glucose synthase (RFFH)	D-Glucose-1-phosphate <=> dTDP-glucose
5.1.3.2	GL30389-R1 GL29575-R1	UDP-glucose 4-epimerase (GALE)	UDP-glucose <=> UD*P*-alpha-D-galactose
2.7.7.13	GL25424-R1	GDP-mannose pyrophosphorylase (GMP)	D-Mannose-1-phosphate - > GDP-mannose

**Table 4:** Key enzymes of EPS biosynthesis in six well-studied mushroom species.

EC No.	*G. lucidum*	*A. cinnamomea*	*C. militaris*	*O. sinensis*	*F. velutipes*	*P. ostreatus*
2.7.1.1	3	2	4	4	2	2
5.3.1.9	1	1	1	1	1	1
5.3.1.8	2	2	1	1	1	1
5.4.2.8	2	0	0	0	0	0
2.7.7.13	1	0	0	0	0	0
4.2.1.47	1	1	0	0	1	1
1.1.1.271	1	1	0	0	1	1
5.4.2.2	1	2	1	1	3	2
2.7.7.9	1	2	1	1	1	1
5.1.3.2	2	1	1	1	1	1
1.1.1.22	1	1	1	1	1	1
4.1.1.35	1	1	0	0	1	1
5.1.3.5	0	0	0	0	0	0
2.7.7.24	0	0	0	0	0	0
4.2.1.46	0	0	1	2	0	0
5.1.3.13	0	0	0	0	0	0
1.1.1.133	0	0	0	0	0	0
2.7.1.6	1	0	1	1	1	1
2.7.7.12	0	0	1	1	1	1
1.1.1.307	1	0	0	0	0	0
1.1.1.9	1	1	2	0	1	1
1.1.1.14	1	1	2	3	3	2
4.1.2.13	2	1	3	2	2	2
3.1.3.11	2	1	1	1	1	1
2.7.1.17	2	2	1	1	2	2
2.2.1.1	0	1	1	1	1	1
2.7.1.11	1	1	1	1	1	1
4.1.2.9	1	1	0	0	1	1
2.7.1.46	1	0	0	0	0	0
2.7.7.64	1	0	0	0	0	0
2.7.1.52	1	0	0	0	0	0
2.7.7.30	1	0	0	0	0	0

*Numbers in table columns are numbers of genes that were annotated.*

#### Identification and Analysis of Essential Genes for EPS Synthesis

Essential genes for EPS synthesis were predicted by single-gene deletion using COBRA Toolbox in two media. Prior to such prediction, biomass function must be constrained to ensure normal growth of cells. For minimal growth medium and fermentation medium, 124 genes (11.70% of total) and 106 genes (10.00%), respectively, were identified as essential for EPS synthesis (Figure 5A; essential genes and simulation conditions are listed in Supplementary Table S4).

For minimal growth medium, predicted essential genes for EPS synthesis were involved primarily in amino acid metabolism (31.45%) and carbohydrate metabolism (21.77%) (Figure 5D). For fermentation medium, predicted essential genes were involved in amino acid metabolism (22.64%), metabolism of cofactors and vitamins (16.04%), lipid metabolism (16.98%, and carbohydrate metabolism (25.47%) (total ∼80%; Figure 5E). These findings indicate that more carbon metabolism pathways are needed for EPS synthesis on minimal growth medium than on fermentation medium.

#### Comparative Genomics Analysis of Nucleoside Sugar Biosynthetic Pathway

To further elucidate EPS metabolic mechanisms and pathways, we performed comparative genomics analysis of *G. lucidum* EPSs and other important fungi.

Genomes of five related edible/ medicinal mushroom species (*Antrodia cinnamomea* (Riley et al., 2014), *Cordyceps militaris* (Zheng et al., 2011), *Ophiocordyceps sinensis* (Hu et al., 2013), *Flammulina velutipes* (Park et al., 2014), *Pleurotus ostreatus* (Riley et al., 2014) were annotated by KAAS. Metabolic enzymes related to EPS biosynthesis in these other species were compared with those in *G. lucidum* to clarify the characteristics of EPS biosynthesis. A total of 32 key enzymes were annotated. Numbers of key enzymes annotated were 25 for *G. lucidum*, 18 for *A. cinnamomea*, 17 for *C. militaris*, 16 for *O. sinensis*, 20 for *F. velutipes*, and 20 for *P. ostreatus* (Table 4). *G. lucidum* and the other five species had comprehensive gene annotations in the glycolysis pathway. All six species displayed gene deletion in the dTDP-rhamnose biosynthetic pathway. For example, dTDP-glucose synthase participated in synthesis of dTDP-glucose, and dTDP-4-dehydro-6-deoxy- D-glucose 3,5-epimerase and dTDP-4-dehydrorhamnose reductase participated in synthesis of dTDP-rhamnose. UDP-arabinose 4-epimerase, which catalyzed conversion of UDP-xylose to UDP-arabinose, was absent.

The above findings, taken together, indicate that the metabolic network of *G. lucidum* is more complex than other fungi, and allows synthesis of a greater variety of fungal nucleoside sugar precursors.

### Optimization Strategies for Improving EPS Production *in silico*

On the basis of our model analysis, we propose two feasible optimization strategies for improvement of EPS production.

**FIGURE 7:**
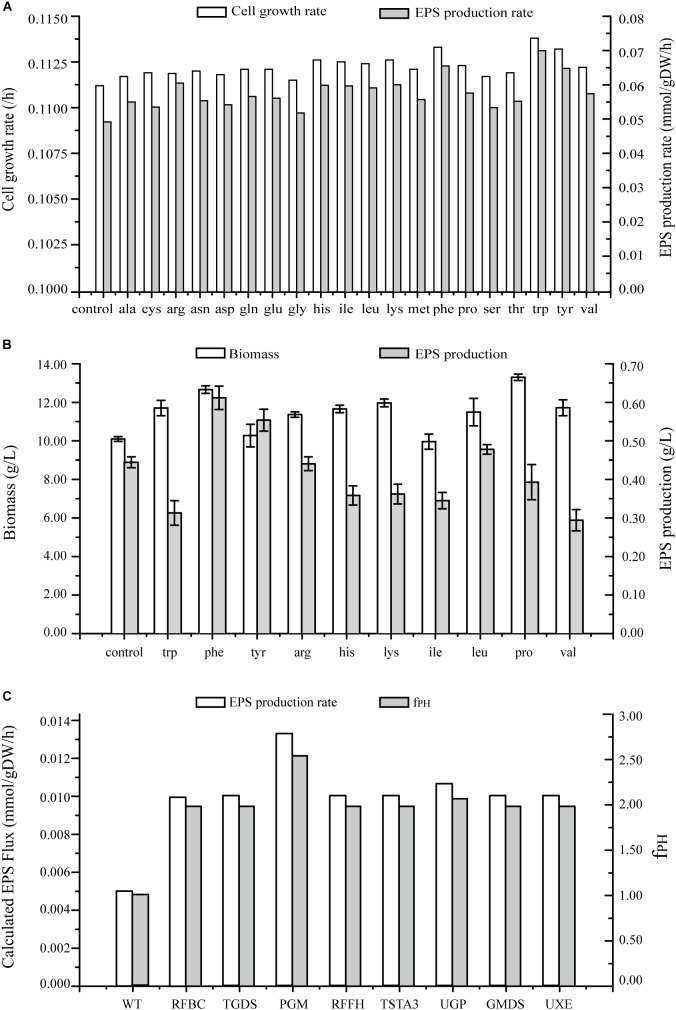
Predicted EPS production rates in *G. lucidum* under various conditions. **(A)** Maximal growth rate and EPS production rate on minimal growth medium, and on minimal growth medium with one of 20 amino acids. **(B)** Effects on biomass and EPS production rate of separate addition of 10 amino acids. **(C)** Effects of single gene overexpression on EPS production rate and f_PH_ (see Simulation, Curation, and Analysis of Model iZBM1060). RFBC, dTDP-4-dehydro-6-deoxy-D-glucose 3,5-epimerase; TGDS, dTDP-glucose 4,6-hydro-lyase; PGM, glucose phosphomutase; RFFH, dTDP-glucose pyrophosphorylase; TSTA3, GDP-fucose synthase; UGP, UDP-glucose pyrophosphorylase; GMDS, GDP-mannose 4,6-hydro-lyase; UXE, UDP-arabinose 4-epimerase.

#### Biochemical Engineering Strategies

Effects of addition of amino acids on cell growth and EPS production were simulated, and both these parameters were found to be enhanced (Figure 7A). EPS production was increased > 15% by addition of 10 separate amino acids in simulation results. Especially, the increase was greatest for tryptophan (41.85%), followed by phenylalanine (32.80%) and tyrosine (31.39%).

The wet experiment results indicated that EPS production was greatly increased by addition of phenylalanine (38.00%) or tyrosine (25.00%) and no enhancement occurred when tryptophan or the other amino acids were applied in EPS production (Figure 7B). Addition of phenylalanine is clearly effective in enhancing EPS production. Polysaccharide yields can be further improved by adjustment of quantity and timing of amino acid addition in future studies.

#### Genetic Engineering Strategies

In several recent studies, expression levels of EPS biosynthetic genes have been manipulated in order to increase EPS production. However, less is known regarding overexpression of target genes for this purpose. On the basis of GSMM, we simulated gene overexpression to guide metabolic engineering for enhancement of EPS production, using MOMA to reevaluate the fluxes and obtain an overexpression algorithm. Eight key enzymes were identified as potential targets for EPS production; i.e., overexpression of PGM gene (EC: 5.4.2.2, GL24280-R1), UGP gene (EC: 2.7.7.9, GL25739-R1), TSTA3 gene (EC: 1.1.1.271, GL21002-R1), GMDS gene (EC: 4.2.1.47, GL20928-R1), UXE gene (EC: 5.1.3.5), RFBC gene (EC: 5.1.3.13), TGDS gene (EC: 4.2.1.46), and RFFH gene (EC: 2.7.7.24) notably enhanced EPS production (Figure 7C and Supplementary Table S5).

PGM catalyzed conversion of glucose-6-P to glucose-1-P; each of these compounds is an important intermediate in EPS biosynthetic pathway. Overexpression of PGM gene increased EPS production from 0.005 mmol/gDW/h in wild-type (WT) to 0.0133 mmol/gDW/h. Thus, increased PGM transcription level was directly correlated with increased EPS production. PGM was also implicated as the key enzyme for EPS biosynthesis in a previous study: maximal EPS production in a PGM gene-overexpressing strain was 1.76 g/L – 44.3% higher than in WT (Xu et al., 2015). Overexpression of UGP gene *in silico* caused an increase of EPS production rate to 0.0106 mmol/gDW/h. UGP is directly involved in synthesis of UDP-glucose, and EPSs contain a high proportion of glucose. UDP-glucose plays a key role in EPS production as a synthetic precursor. Li et al. (2015) also demonstrated an effect of UGP on EPS synthesis.

UXE catalyzes interconversion of two EPS synthesis precursors: UDP-arabinose and UDP-xylose. When glucose is used as carbon source, UXE plays an essential role in production of UDP-arabinose. TSTA3 and GMDS are involved in synthesis of GDP-fucose. RFBC, TGDS, and RFFH participate in synthesis of dTDP-rhamnose.

Results of the analysis described above indicate that EPS production can be effectively improved by overexpression of genes for eight key enzymes. Previous studies have demonstrated the usefulness of PGM and UGP genes in this regard. Future studies will focus on overexpression of the other six genes for improvement of EPS production.

## Conclusion

A GSMM for *Ganoderma lucidum* (lingzhi mushroom) is presented here for the first time. The GSMM (termed model *i*ZBM1060) is focused on EPSs, and contains 1404 reactions, 1202 metabolites, and 1060 genes. The model was validated and shown to accurately simulate cell growth and EPS production under various conditions. The nucleoside sugar (EPS precursor) biosynthetic pathway in the model was elucidated completely. Essential genes for cell growth and EPS synthesis, and genes for eight key EPS production enzymes, were analyzed. Two strategies for improvement of EPS production, based on model *i*ZBM1060, were proposed: (i) addition of phenylalanine; (ii) overexpression of the eight key enzyme genes. PGM and UGP genes have previously been shown to be useful targets for enhancement of EPS production, and future studies will focus on overexpression of the other six genes for this purpose. Model iZBM1060 provides a useful platform for regulating EPS production in terms of system metabolic engineering for *G. lucidum*, as well as a guide for future metabolic pathway construction of other high value-added edible/ medicinal mushroom species.

## Author Contributions

ZM and ZD designed the experiments. ZM, WD, QW, and MX performed the experiments. ZM, CY, GL, FW, GS, ZD, LL, and ZX conceived the project, analyzed the data, and wrote the paper.

## Conflict of Interest Statement

The authors declare that the research was conducted in the absence of any commercial or financial relationships that could be construed as a potential conflict of interest.

## Abbreviations

Ac: acetate
Acal: acetaldehyde
Accoa: acetyl-CoA
Akg: alpha-2-oxoglutarate
Aol: D-arabinitol
Ara: arabinose
Ara1p: beta-arabinose-1-P
Cellob: cellobiose
Cit: citrate
D6pgc: 6-*P*-gluconate
D6pgl: 6-*O*-*P*-glucono-1,5-lactone
dTDP-Glc: dTDP-alpha-glucose
dTDP-Oglc: dTDP-4-oxo-6-deoxy-glucose
dTDP-Rmn: dTDP-4-dehydro-beta-rhamnose
E4p: erythrose-4-P
Eth: ethanol
Fdp: fructose-1,6-bisphosphate
Fru: fructose
Fuc: fucose
Fuc1p: fucose-1-P
Ful: fuculose
Fulp: fuculose-1-P
Fum: fumarate
G: glycerate
Ga3p: glyceraldehyde-3-P
Gal1p: alpha-galactose-1-P
GDP-Ddm: GDP-4-dehydro-6-deoxy-mannose
GDP-Fuc: GDP-fucose
GDP-Man: GDP-mannose
Gl: glycerol
Gl3p: sn-glycerol-3-P
Glac: galactose
Glx: glyoxylate
Glyal: glyceraldehyde
Glyn: glycerone
Icit: isocitrate
Kdr: 2-dehydro-3-deoxy-rhamnonate
Lact: lactose
Laol: L-arabinitol
Lxul: L-xylulose
Mal: malate
Man: mannose
Man1p: mannose-1-P
Man6p: mannose-6-P
Meli: melibiose
Mlt: maltose
Oaa: oxaloacetate
13Pdg: 1,3-bisphosph-glycerate
2pg: 2-*P*-glycerate
3pg: 3-*P*-glycerate
Pep: phosphoenolpyruvate
Pyr: pyruvate
R5P: ribose-5-P
Rl: ribulose
Rl5p: ribulose-5-P
Rmf: rhamnofuranose
Rml: rhamnono-1,4-lactone
Rmn: rhamnose
Rmna: rhamnonate
S7p: sedoheptulose-7-P
Suc: sucrose
Succ: succinate
Succoa: succinyl-CoA
T3p2: glycerone-P
Tre: trehalose
UDP-Ara: UDP-arabinose
UDP-Ga: UDP-glucuronate
UDP-Glac: UDP-alpha-galactose
UDP-Glc: UDP-glucose
UDP-Xyl: UDP-xylose
Xol: xylitol
Xul: D-xylulose
Xul5p: xylulose-5-P
Xyl: xylose.
