# Supplementary material for: Reconstruction and Analysis of a Genome-Scale Metabolic Model of Ganoderma lucidum for Improved Extracellular Polysaccharide Production
Source: Front Microbiol. 2018 Dec 11;9:3076. doi: 10.3389/fmicb.2018.03076 (PMC6298397; doi:10.3389/fmicb.2018.03076)
Supplement: Supplementary file 6 [file Data_Sheet_1.docx]

**Supplementary Material**

**Reconstruction and analysis of** **a genome-scale metabolic model of *Ganoderma lucidum* for improved** **extracellular polysaccharide production**

**Zhongbao Ma****^1, 2^, Chao Ye^1, 2^, Weiwei Deng^2^, Mengmeng Xu****^1, 2^, Qiong Wang^1, 2^, Gaoqiang Liu****^3^, Feng Wang^4^, Liming Liu^1, 2^, Zhenghong Xu****^1, 2^, Guiyang Shi^1,^** **^2^, Zhongyang Ding^1, 2, *^**

^1^ Key Laboratory of Carbohydrate Chemistry and Biotechnology, Ministry of Education, School of Biotechnology, Jiangnan University, Wuxi 214122, China.

^2^ National Engineering Laboratory for Cereal Fermentation Technology, Jiangnan University, Wuxi 214122, China.

^3^ Key Laboratory of Cultivation and Protection for Non-Wood Forest Trees, Ministry of Education, College of Life Science and Technology, Central South University of Forestry and Technology, Changsha 410004, China.

^4^ School of Food and Biological Engineering, Jiangsu University, Zhenjiang 212013, China.

^*^ **Correspondence:**

Dr. Zhongyang Ding

bioding@163.com

**Supplementary** **figure**


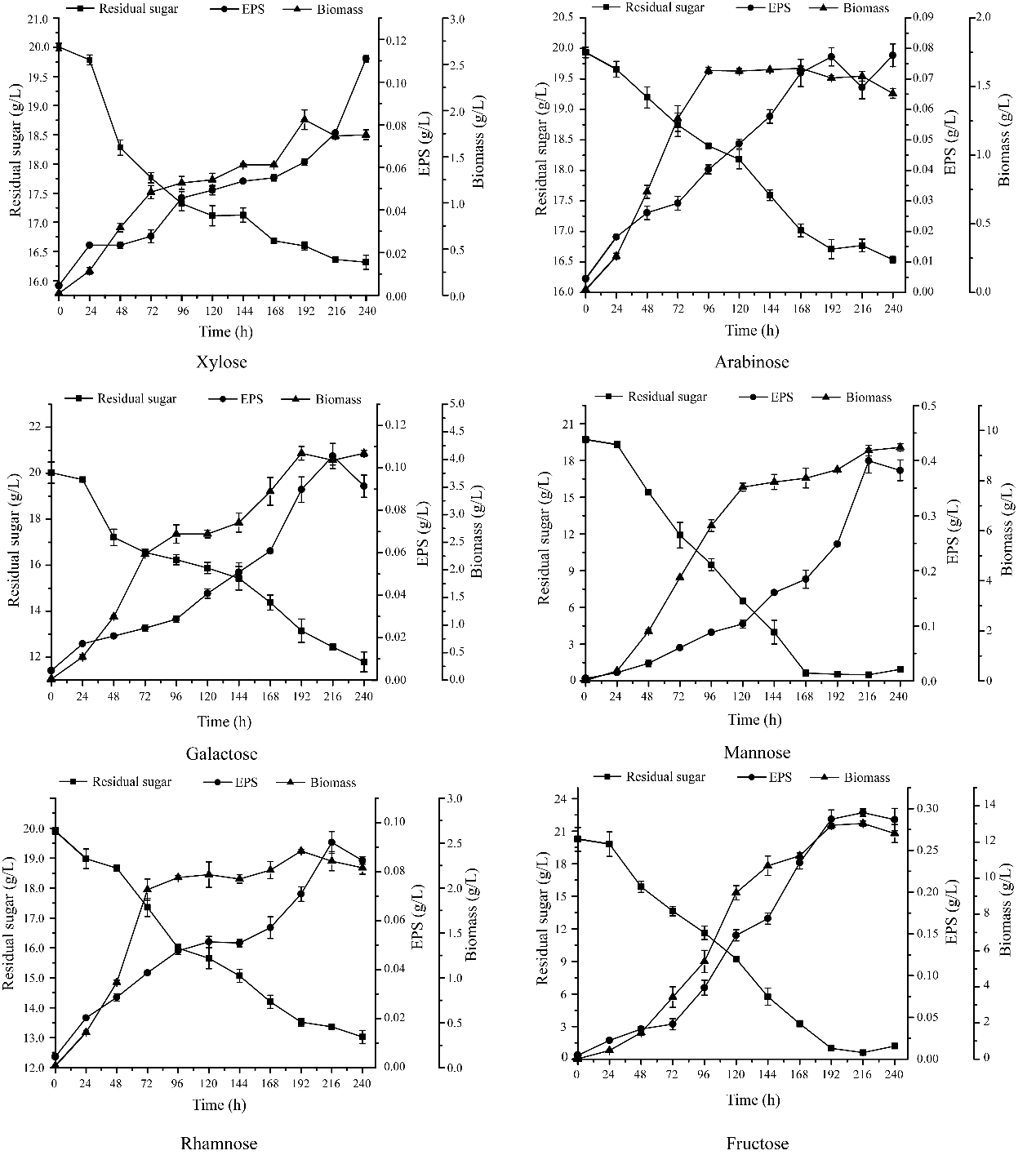


**Figure S1.** Effects of six carbon sources on biomass and EPS production.
